# Exercise Stress Testing in Clinical Cardiology: A Practical Guide to Performance and Interpretation

**DOI:** 10.3390/jcm15041656

**Published:** 2026-02-22

**Authors:** Chiara Carluccio, Francesco Bressan, Matteo Pizzolato, Amedeo De Antoni, Simone Ungaro, Dorottya Balla, Alberto Cipriani, Manuel De Lazzari, Martina Perazzolo Marra, Hajnalka Vago, Domenico Corrado, Alessandro Zorzi, Francesca Graziano

**Affiliations:** 1 Department of Cardiac, Thoracic, Vascular Sciences and Public Health, University of Padua, Via Giustiniani 2, 35128 Padua, Italy; chiara.carluccio@studenti.unipd.it (C.C.); francesco.bressan.2@studenti.unipd.it (F.B.); amedeo.deantoni@studenti.unipd.it (A.D.A.); simone.ungaro@studenti.unipd.it (S.U.); alberto.cipriani@unipd.it (A.C.); manuel.delazzari@aopd.veneto.it (M.D.L.); martina.perazzolomarra@unipd.it (M.P.M.); domenico.corrado@unipd.it (D.C.); alessandro.zorzi@unipd.it (A.Z.); 2 Heart and Vascular Center, Semmelweis University, 1122 Budapest, Hungary; balla.dorottya@semmelweis.hu (D.B.); vago.hajnalka@semmelweis.hu (H.V.); 3 Department of Sports Medicine, Semmelweis University, 1122 Budapest, Hungary

**Keywords:** exercise stress testing, functional capacity, inducible ischemia, arrhythmias, premature ventricular beats, ion channel disease, ventricular pre-excitation

## Abstract

Exercise stress testing remains one of the most widely used and cost-effective diagnostic tools in clinical cardiology. Beyond the traditional evaluation of induced ischemia, it provides valuable information on functional capacity, blood pressure response and arrhythmic behavior during exercise. In particular, the test plays a crucial role in assessing and interpreting exercise-induced arrhythmias, including tachyarrhythmias, such as premature ventricular beats (PVBs) and bradyarrhythmias, as well as corroborating the suspicion of some ion channel diseases. The usefulness of exercise testing is also highlighted in patients with devices, where it can help evaluate their function and exercise adaptation, as well as in specific conduction disorders, such as Wolff–Parkinson–White syndrome. This practical guide summarizes the key aspects of performing and interpreting the exercise stress test, focusing on hemodynamic and arrhythmic findings and their clinical implications, and includes several illustrative clinical cases.

## 1. Introduction

Exercise stress testing (EST) has been used for decades as a non-invasive procedure to provide diagnostic and prognostic information in patients with known or suspected heart disease. The procedure entails continuous 12-lead electrocardiographic monitoring while the patient undergoes physical exercise, typically on a treadmill or cycle ergometer, according to standardized protocols. Concomitantly, blood pressure is monitored at regular intervals, and clinical symptoms are recorded.

EST is the most widely available functional test, relatively low-cost and easy to administer. Traditionally, EST has been employed to assess for inducible ischemia in patients with suspected coronary artery disease (CAD) [1,2,3,4]. However, its diagnostic accuracy for detecting obstructive CAD is modest. Beyond ischemia detection, EST represents a fundamental clinical tool to provide information on exercise capacity, chronotropic and blood pressure (BP) response to exercise, detection of exercise-induced arrhythmias and conduction disorders, to assess the effectiveness of cardiovascular (CV) therapy and exercise response in patients with cardiac implantable electronic devices (CIEDs) [4,5].

This review examines the physiological basis of exercise, EST methodologies, the indications and specific clinical applications and provides a practical, easy-to-consult guide to EST performance and interpretation.

## 2. Physiology of Cardiovascular Response to Exercise

The ability to perform physical exercise is enabled by a progressive increase in oxygen uptake (VO_2_). Maximal oxygen uptake (VO_2_ max) is defined as the greatest amount of oxygen a subject can consume during dynamic exercise and represents an excellent measure of CV fitness and functional capacity. The amount of oxygen required during rest, per unit mass of 1 Kg body weight and time, is defined as the metabolic equivalent (MET) and is equal to 3.5 mL O_2_/Kg/min. Functional capacity is typically expressed as estimated METs, which represent multiples of the basal rate of oxygen consumption at rest [6,7,8].

VO_2_, as defined by the Fick equation, is the product of cardiac output and peripheral arteriovenous oxygen difference. During the initial phases of exercise, the increase in cardiac output is driven by an increase in peripherical oxygen extraction, a rise in stroke volume (mediated through the Frank–Starling mechanism) and by an increase in HR. Conversely, during maximal aerobic exercise, the continued rise in VO_2_ is mainly due to an increase in HR, as stroke volume reaches a plateau at 50–60% of VO_2_ max. HR is, at any moment, the result of a dynamic balance between sympathetic and parasympathetic nervous system influences [8,9]. In healthy subjects, an average resting HR of 60–80 beats per minute (bpm) reflects a basal predominance of parasympathetic tone. As exercise progresses, the increase in sympathetic discharge and the inhibition of parasympathetic stimulation result in a positive chronotropic and inotropic response, leading to a progressive increase in HR and myocardial contractility. At submaximal workloads below the lactate or ventilatory threshold (i.e., the exercise intensity at which lactate production and buffering begin to exceed clearance capacity), HR, cardiac output, blood pressure, and ventilation increase proportionally to workload and remain physiologically regulated. Although anaerobic metabolism contributes at all exercise intensities, this threshold reflects the point at which glycolytic pathways become progressively predominant, leading to systemic lactate accumulation and a disproportionate increase in ventilation. As exercise intensity surpasses this threshold, sympathetic discharge further increases, promoting peripheral vasoconstriction in most vascular beds (except exercising muscle and the cerebral and coronary circulations) and enhanced oxygen extraction [7]. In patients with heart failure, reduced beta-adrenergic receptor density and desensitization may contribute to chronotropic incompetence and impaired exercise capacity. Nevertheless, VO_2_ peak reduction is multifactorial and phenotype-dependent, reflecting both central hemodynamic limitations and peripheral abnormalities in oxygen extraction [8].

The maximal HR achieved during exercise is strongly influenced by age and age-related hormonal and autonomic factors, although recent evidence suggests a substantial heritable genetic component [10,11]. The maximal HR (HR max) could be predicted from one of several available equations. The most straightforward and commonly used equation [predicted HR max = 220 − age in years] is burdened by high intersubject variability and tends to overestimate HR max in younger women but tends to underestimate it in older women. Newer equations have been proposed to predict the HR max more accurately [7,12]:

Men: HR max = 208 − (0.7 × Age)

Women: HR max = 206 − (0.88 × Age)

Alternatively, exercise intensity can be expressed as a percentage of a person’s HR reserve (predicted maximum HR − resting HR) using the Karvonen formula [5].

Immediately after exercise interruption, sympathetic withdrawal and increased parasympathetic tone cause a rapid decline in HR, defined as HR recovery (HRR). In routine clinical practice, HRR is conventionally assessed at 1 min after exercise cessation, and less frequently at 2 min. In contrast, earlier time points (e.g., 10–30 s) are primarily used in research settings and in athletic populations to better characterize autonomic reactivation. Highly trained athletes often exhibit a rapid drop in HR of 30–50 bpm during the first minute of recovery due to parasympathetic overactivity [9,13,14]. Conversely, the Multiple Risk Factor Intervention Trial (MRFIT) demonstrated that a delayed HR recovery (<50 bpm after 3 min) was an independent predictor of all-cause death in asymptomatic men [15]. It was recently reported that decreased HR recovery at 10 s after cessation of exercise is a superior predictor of outcome compared with the same indicator at later time intervals [16]. Generally, the HR should decrease by at least 12 beats in the first minute of recovery [17].

HR variability during exercise and recovery from exercise is an important physiological marker reflecting vagal and sympathetic nerve activity. If high HR variability is associated with healthy conditions, low HR variability reflects an underlying pathological substrate [18]. As a result, imbalances in autonomic control of CV activity both during and after exercise are strongly associated with increased risk of adverse CV outcomes and sudden death [15,19,20].

Under physiologic conditions, systolic blood pressure (BP) increases linearly with exercise intensity, reflecting increased cardiac output to meet metabolic demands, whereas diastolic pressure usually remains stable or is moderately decreased because of vasodilatation of the vascular bed [7]. BP usually increases by 5–10 mmHg/MET [21] during a progressive exercise test, while diastolic BP exhibits little or no change (<10 mmHg) due to peripheral vasodilatation. Moreover, in healthy and highly trained individuals, the more pronounced CV adaptation to intensive long-term endurance training leads to a greater increase in cardiac output, with a greater increase in volume overload and systolic BP during exercise, while diastolic BP usually remains unchanged due to extensive peripheral vasodilation [22,23].

Following maximal exercise, systolic BP rapidly declines as cardiac output decreases, usually reaching resting levels or lower within 6 min. However, stopping exercise abruptly can cause precipitous drops in pressure due to venous pooling and a delayed increase in vascular resistance. To mitigate these hemodynamic risks, an active cool-down period is strongly recommended [7]. Several factors, including age, sex, sex-related hormonal modulation, cardiorespiratory fitness, training level, and cardioactive medication, influence BP response to exercise [24,25,26]. An exaggerated hypertensive response or an inadequate/hypotensive SBP response during exercise has been associated with masked hypertension and an increased risk of future CV events, although no universally accepted definition is currently provided in European hypertension guidelines [24].

The index derived from the product of HR and systolic BP obtained during graded exercise is defined as Rate-Pressure Product (RPP) or Double Product. It is a derived hemodynamic index that serves as a non-invasive surrogate of myocardial oxygen consumption (MVO_2_). In healthy subjects, RPP increases progressively with exercise intensity, reflecting the augmenting cardiac work required to meet peripheral metabolic needs. Peak RPP values in healthy individuals typically range between 20,000 and 35,000 mmHg·bpm. This peak value characterizes the maximal aerobic performance of the CV system. By contrast, a failure to raise the systolic BP or HR adequately (low peak RPP) is associated with increased risk of adverse CV events and poor hemodynamic reserve [7].

## 3. Indications and Contraindications of Exercise Stress Testing

The broad indications of EST encompass diagnosis, prognosis, functional assessment, and evaluation of therapeutic interventions.

Exercise stress testing is recommended for assessing exercise tolerance and inducible ischemia during the initial diagnostic evaluation of patients with suspected chronic coronary syndrome [4,5,27,28]. In asymptomatic master athletes, although EST is a poor predictor of CAD, it could help identify previously unrecognized medical conditions such as hypertension or exercise-induced ventricular arrhythmias, guiding subsequent clinical management [29,30].

Although CPET remains the preferred tool for comprehensive evaluation of exercise physiology in patients with heart failure and cardiomyopathy, EST may still be considered in selected cases for functional assessment and arrhythmic risk evaluation, particularly when CPET is not available [5].

EST is indicated for detecting exercise-induced arrhythmias, diagnosing chronotropic incompetence, frequency-induced atrioventricular and bundle branch blocks, and assessing exertional symptoms in both sedentary individuals and athletes during pre-participation screening [31]. It is also recommended for the diagnosis and assessment of therapy response in patients with suspected or proven adrenergic-dependent rhythm disturbances (e.g., catecholaminergic polymorphic ventricular tachycardia) [32,33]. In addition, assessment of the QTc interval during exercise and recovery may aid in the diagnosis of long QT syndrome (LQTS) [33]. It is also widely used as a non-invasive tool for risk stratification in patients with asymptomatic ventricular pre-excitation.

It can diagnose CIED-mediated chronotropic incompetence and provide HR targets to optimize pacemaker programming.

The specific applications of EST will be detailed in the following sections. Common contraindications are listed in Table 1 [7].

### How to Define a Maximal Exercise Testing and When to Stop It

Historically, the indications for EST termination were established based on populations with ischemic heart disease. In this clinical context, an HR threshold of 85% of Maximal Theoretical Heart Rate (MTHR) was set to ensure safe training below the individual ischemic threshold. Consequently, this cut-off became the standard parameter for defining an EST “maximal” in pathological conditions. Nevertheless, the indications for EST terminations in subjects without known CV disease are not unequivocally established. Evidence suggests that, in healthy individuals, 85% of MTHR is ineffective at quantifying patients’ maximal exertion during EST [34]. Sirico et al. demonstrated that the majority of healthy subjects exceeded 85% of MTHR, reporting only moderate perceived exertion (typically around 14 in the Rating of Perceived Exertion Borg Scale), indicative of submaximal effort. Moreover, almost half of the recorded ECG events (both ischemic and arrhythmic) occurred at HR > 85% of MTFR; thus, terminating EST at 85% of MTHR limits diagnostic accuracy in detecting ECG abnormalities [35]. Therefore, EST is defined as maximal when performed until physical exhaustion, as indicated by a Rating of Perceived Exertion Scale > 17 [35]. In patients receiving beta-blocker therapy, the negative chronotropic effect may blunt the HR response to exercise, preventing the attainment of 85% of MTHR. However, if the EST is conducted until physical exhaustion, it can be considered maximal and diagnostic.

By contrast, in deconditioned patients, an exaggerated HR response to physical exertion may occur early; also, in this case, EST should continue until symptom-limited exertion.

EST should be terminated at the onset of typical angina, ST-segment elevation (>1.0 mm in leads other than aVR, aVL, or V1, and without Q waves due to prior myocardial infarction), or complex ventricular arrhythmias with hemodynamic compromise. Further absolute and relative indications for EST termination are listed in Table 2 [7]. Nonetheless, the safety of exercise testing is well established, and the overall risk of adverse events is low, though it depends on the study population’s characteristics [7].

## 4. Exercise Testing Procedure

### 4.1. Patient Preparation

Before performing an EST, a comprehensive clinical assessment is essential to confirm the indication for testing, to ensure the appropriateness of the selected protocol, evaluate the patient’s ability to perform exercise, and identify any contraindications (Table 1). In selected patients with unexplained symptoms, suspected structural heart disease, significant valvular abnormalities, cardiomyopathy, or aortic pathology, transthoracic echocardiography should be considered prior to exercise testing to better define baseline cardiac structure and function. The pre-test evaluation should integrate information from the patient’s medical history supplemented by a focused physical examination. A current resting 12-lead ECG is crucial to assess HR, rhythm, conduction abnormalities, and evidence of prior myocardial infarction, and, when available, it should be compared with previous ECGs. Resting BP measurement should be performed to screen for baseline hypertension/hypotension and assess the safety and feasibility of the EST.

Patients should be advised to wear comfortable exercise clothing and supportive footwear. Dietary restrictions include abstaining from significant food intake for two to three hours before testing, while adequate hydration is encouraged. Furthermore, patients must avoid caffeine, alcohol, and tobacco before the procedure [7].

Medication management should be tailored to the clinical objective. It is recommended to continue usual therapy when EST is performed to evaluate the efficacy of cardioactive drugs, such as for controlling exercise-induced arrhythmias, BP response to exercise, or to determine their functional and hemodynamic effects. Conversely, cardioactive drugs (especially β-blockers) should be withheld for 24 h prior to the EST if the purpose is to evaluate the exercise response without medication. In patients with a diagnosis of CAD, cardioactive drugs should generally be maintained.

In patients with CIEDs, device type, programming, rate responsiveness, and pacing limits must be reviewed in advance. Specifically, for patients with implantable cardioverter-defibrillators (ICDs), information about ICD rhythm detection and therapy thresholds should be identified to ensure that peak exercise HR remains safely below programmed intervention zones [12]. If possible, continuous device telemetry monitoring should be performed during the test to verify appropriate sensing and rhythm discrimination at increasing workloads. These precautions apply regardless of the underlying cardiac disease.

Before EST, the patient should be familiarized with the symptom rating scales used to monitor perceived effort and symptoms during the procedure. The most used are the Borg Rating of Perceived Exertion (RPE) and the Borg Category-Ratio 10 (CR10) scale. The Borg RPE scale ranges from 6 to 20, with each number corresponding to the perceived exertion, from 6 (no exertion at all) to 20 (maximal exertion). The scale was initially designed to correspond to HR when multiplied by 10. The Borg CR10 scale ranges from 0 (no exertion) to 10 (maximal exertion) [12].

During EST, ECG is monitored continuously. To obtain a high-quality 12-lead ECG acquisition, meticulous skin preparation, including hair removal and mild abrasion of the superficial epidermal layers, is required.

**Figure 1:**
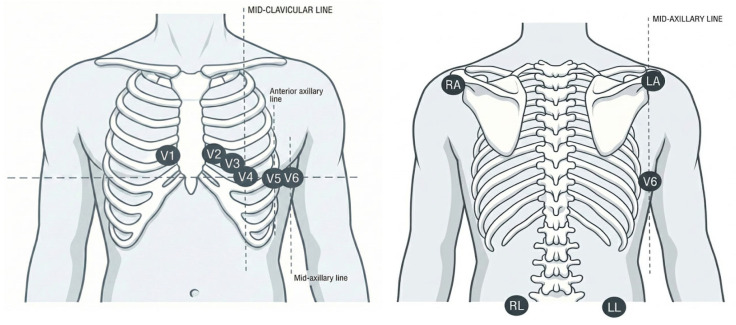
Representation of lead placement for noise reduction during ECG-EST. LA, left arm; LL, left leg; RA, right arm; RL, right leg.

Furthermore, the Mason–Likar modification of the standard 12-lead ECG is routinely employed to reduce motion-induced artifacts by relocating the limb electrodes to the torso. In this configuration, the arm electrodes are positioned at the distal aspects of the infraclavicular fossae, while the leg electrodes are placed on stable sites above the iliac crests (Figure 1).

Blood pressure should be assessed at baseline, at 2 min intervals during exercise or according to necessity, and at least twice during the recovery period. During exercise stress testing, blood pressure measurement should preferably be performed manually using the auscultatory method, particularly during treadmill protocols, since motion artifacts and patient movement may reduce the reliability of non-validated oscillometric devices. Automated devices specifically validated for exercise conditions can be acceptable, especially during cycle ergometer testing, but abnormal readings should be confirmed manually [36].

### 4.2. Exercise Testing Room

**Figure 2:**
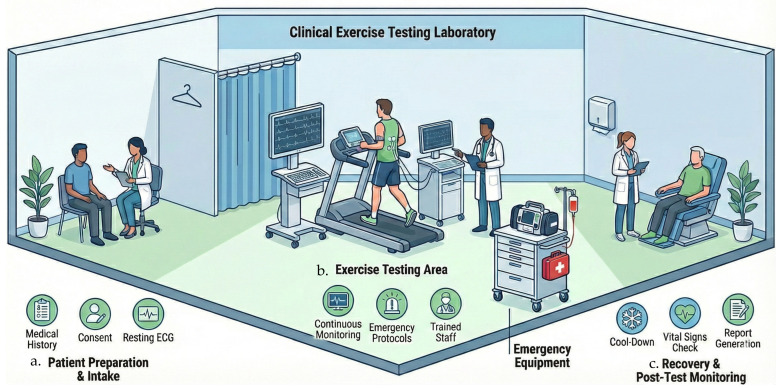
Schematic representation of a clinical exercise testing laboratory. (**a**) Patient intake and preparation, resting ECG acquisition. (**b**) Supervised exercise testing. (**c**) Post-test recovery with vital signs assessment and report generation.

The testing room should be large enough to ensure patient privacy and provide adequate space around exercise ergometers for staff movement and immediate access for emergency equipment. Climate control is essential to prevent heat-related complications; the guidelines recommend maintaining a cool temperature (ideally 20 °C to 22 °C) and adequate ventilation [7,37]. Crucially, a fully equipped emergency crash cart containing appropriate drugs and a defibrillator must be immediately accessible. Figure 2 schematically illustrates the characteristics of a testing room and the patient assessment process.

### 4.3. Exercise Testing Protocols and Modality

Treadmill and cycle ergometer testing are the primary modalities for dynamic EST in clinical practice. The choice of testing modality and protocol should be guided by the patient’s estimated functional capacity, considering factors such as age, physical fitness, and underlying disease.

Treadmill and cycle ergometer EST can be conducted using either stepped or continuous ramp protocols. Stepped protocols typically increase work rate by 1 to 2.5 METs per stage. Ramp protocols, in contrast, use smaller, constant-workload increments with stages lasting no longer than 1 min, aiming for the patient to achieve peak exercise capacity within 8 to 12 min [37].

The most used stepped treadmill protocols are the Bruce, modified Bruce, and Naughton protocols, which offer standardized workload progression for a broad spectrum of functional capacities. The metabolic cost in METs of the treadmill work rate can be estimated from the speed and grade of elevation using standardized equations [12,38]. The standard Bruce treadmill protocol, first described by Robert Bruce in 1973 [39], is widely used in healthy individuals. It is a maximal, multistage protocol consisting of 3 min stages, designed to allow the achievement of steady-state conditions before the workload increases. Compared with the ramp protocol, the Bruce protocol exhibited higher sensitivity for detecting myocardial ischemia, owing to higher HR and a higher Double Product at peak exercise, favoring its use in screening settings [40]. In older adults and in patients with cardiac-related exercise limitations, a modified version including two initial 3 min warm-up stages at 2.4 km/h with 0% and 5% incline is commonly employed. A major limitation of the Bruce protocol is the relatively large increment in VO_2_ between stages and the increased energetic cost of running in later stages. In contrast, the Naughton and Weber protocols, which use shorter stages (1–2 min) with smaller workload increments of approximately 1 MET per stage, are better suited for patients with reduced exercise tolerance, including those with stable chronic heart failure [12].

Cycle ergometry is a suitable alternative to treadmill testing, especially for patients with orthopedic, peripheral vascular, or neurological impairments that limit weight-bearing capacity. The cycle ergometer EST utilizes incremental workloads calibrated in watts (W) or kilogram-meters per minute (kg·m/min), with 1 W equivalent to approximately 6 kg·m/min. In mechanically braked cycle ergometers, workload is determined by the applied resistance and pedaling distance, necessitating that the patient maintain a constant cadence, typically 60–80 revolutions per minute (rpm). Electronically braked cycle ergometers, by contrast, maintain a constant workload regardless of variations in pedaling cadence and are therefore less reliant on patient cooperation. Most cycle ergometer protocols commence at a workload of 10 or 25 W (approximately 150 kg·m/min), with incremental increases of 25 W every 2 or 3 min until predefined or symptom-limited endpoints are achieved. For younger or more physically fit individuals, protocols may begin at 50 W, with subsequent increments of 50 W every 2 min. Ramp protocols differ from stepped protocols by initiating with some minutes of unloaded pedaling, followed by a continuous and uniform increase in workload, typically by 5 to 30 W per minute, based on the patient’s anticipated exercise capacity [41,42].

Regardless of the specific protocol chosen, EST should be individualized to achieve a fatigue-limited exercise duration of ≈8 to 12 min. Any EST duration of less than 8 min typically results in a 10% reduction in maximal VO_2_, with a nonlinear relationship between VO_2_ and work rate. By contrast, a protocol exceeding 12 min may lead to test termination due to muscle fatigue or orthopedic factors rather than cardiopulmonary end points [43,44]. Accurate prediction of peak work rate is important to bring subjects to their maximal performance within the recommended 8–12 min; several predictive equations have been developed to estimate peak work rate using weight, height, sex, and ethnicity as variables; however, none of these is adapted and validated to the subject’s fitness and training level [45,46]. For this reason, the expertise of the clinicians in individualizing the right protocol, given the fitness level of the patient, is essential. Indeed, in high-performance athletes, longer or sport-specific protocols may be required to accurately characterize exercise capacity and to inform individualized training recommendations. In selected endurance disciplines, lactate steady-state testing may be necessary to precisely define training intensity domains.

Physiological demands vary significantly between testing modalities; notably, VO_2_ max achieved with cycle ergometer EST is generally reduced by 5% to 20% compared to treadmill protocols [7]. When serial testing is performed for follow-up or training monitoring, the same testing modality and protocol should be maintained to ensure valid intraindividual comparison.

## 5. Exercise Testing in Clinical Practice

### 5.1. Inducible Ischemia

EST has long been a key non-invasive tool in traditional clinical cardiology for assessing inducible ischemia in patients with suspected CAD. However, its diagnostic accuracy for detecting CAD is lower than that of modern functional or anatomical imaging modality, with sensitivity and specificity ranging from 60% to 77%. Nevertheless, EST remains a valuable method for prognostic assessment and risk stratification [47,48,49].

Exercise stress testing is recommended for assessing inducible ischemia during the initial diagnostic evaluation of patients with suspected chronic coronary syndrome. It is particularly useful for individuals with a low (5–15%) pre-test likelihood of obstructive CAD, as it can facilitate reclassification into the very low likelihood category, in whom further testing can be deterred [4,27].

Moreover, several position statements recommend CV screening with EST in athletes over 35 years and in sedentary individuals at high CV risk who plan to undertake high-intensity exercise in order to early detect CAD, potentially at risk of sudden cardiac death [5,28].

Exercise-induced angina is a key predictor of the presence and severity of CAD. Christman et al. [50] demonstrated that exercise-induced typical angina, occurring during EST, independently predicts CV events, irrespective of ECG abnormalities.

During EST, ST-T changes may suggest CAD and warrant further evaluation.

**Figure 3:**
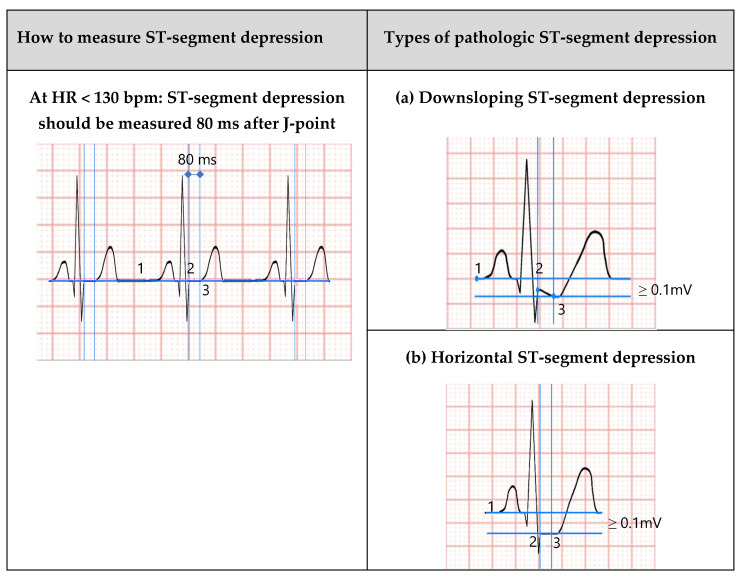

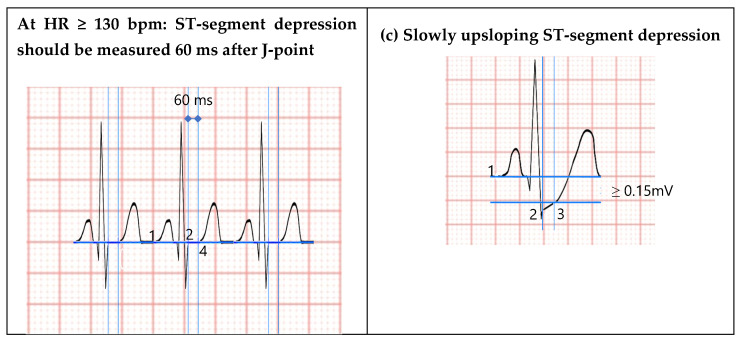
Definition of ST-segment depression changes during exercise. On the left column, example of how to correctly measure ST-segment depression. On the right column, abnormal ST-segment responses to EST: horizontal (**a**) and downsloping (**b**) ST-segment depression ≥ 1 mV and slowly upsloping (0.5–1.0 mV/s) ST-segment of >0.15 mV (**c**). Define the isoelectric line (1); define the j-point (2); measure the ST-segment depression after 80 msec from the j-point (3), or 60 msec (4) according to the heart rhythm (HR).

The usual criterion to define pathological ST-segment depression is the presence of a horizontal (0.7–1 mV/s) or downsloping ST depression of 0.10 mV (1 mm) or greater in at least three consecutive beats. ST-segment evaluation should be read at 80 ms from the J-point and at 60 ms from the J-point when HR is greater than 130 bpm (Figure 3) [51].

J-point depression is a normal finding during maximal exercise. Rapid upsloping ST depression (>1 mV/s) of <0.15 mV, read 80 ms after the J-point, is usually a normal response to exercise and gradually returns to pre-exercise values during recovery [7]. Conversely, slowly upsloping (0.5–1.0 mV/s) ST-segment depression of >0.15 mV is abnormal and is typically seen in patients with known obstructive CAD [48,51].

ST-segment depression observed exclusively during the recovery phase of EST has diagnostic and prognostic significance comparable to that observed during the active exercise phase [52]. Furthermore, the persistence of ST-segment changes beyond 1 min of recovery is tied to a worse prognosis and more extensive CAD. Conversely, the resolution of ECG abnormalities within 1 min of recovery indicates a lower likelihood and severity of obstructive CAD [53].

The earlier ST-segment depression appears during EST and the longer it persists into recovery, the greater the probability of CAD.

Exercise-induced ST elevation of 0.1 mV (1 mm) or greater in at least three consecutive beats is a marker of inducible ischemia. In particular, the presence of ST-segment elevation in lead aVR is a sensitive predictor of left main CAD or multivessel CAD [54,55].

In individuals with Wolff–Parkinson–White (WPW) syndrome, abnormal ventricular activation and repolarization, due to the accessory pathway, frequently lead to ST-segment depression or T-wave inversion, which can be misinterpreted as ischemic changes [56]. Furthermore, chronic treatment with digitalis glycosides can compromise ST-segment assessment. Digitalis administration typically induces a prominent J-point depression and, less commonly, ST-segment depression. Notably, these changes are often evident at rest but can be accentuated during exercise [57]. Therefore, ECG-EST evaluation in patients taking digitalis is associated with a higher risk of false-positive results and reduced diagnostic value.

When evaluating exercise-induced ST-segment depression, it should be recognized that exaggerated atrial repolarization waves during exercise may produce an apparent downsloping ST depression, particularly in inferior leads, even without underlying ischemia. Consequently, isolated inferior ST-segment depression is frequently a false positive resulting from atrial repolarization artifacts [58].

The presence of resting ECG abnormalities prevents ST-segment evaluation during EST; these conditions include LBBB, ventricular pacing, ventricular pre-excitation, and resting ST depression ≥ 0.1 mV. In patients with established CAD, the test may still be considered to assess functional status and symptom onset, complementing the overall clinical assessment [4].

### 5.2. Functional Capacity

As explained in Section 2, functional capacity is a strong predictor of mortality and nonfatal CV outcomes in both patients with and without CAD.

It was reported that each 1-MET increase in functional capacity is associated with a 17–20% reduction in the risk of CV mortality, regardless of the indication for EST [59].

Predicted functional capacity adjusted for age and sex could be estimated by a simple regression equation [7]: predicted METs = 18 − (0.15 × Age) in men or 14.7 − (0.13 × Age) in women.

The two fundamental physiologic parameters that provide relevant information concerning functional capacity and prognosis are HR and BP responses to exercise.

#### 5.2.1. Chronotropic Incompetence and Heart Rate Recovery

Chronotropic incompetence is defined as the inability of the heart to increase its rate adequately to meet the body’s metabolic demand during exertion. This condition significantly contributes to exercise intolerance and is an independent predictor of CV events and mortality. Failure to achieve maximal predicted HR, inadequate submaximal HR, or HR instability during exertion are all examples of impaired chronotropic response [8]. In patients with HF, the mechanism underlying chronotropic incompetence involves reduced β-adrenergic receptor density and sensitivity secondary to increased sympathetic drive [8,60,61]. Chronotropic incompetence is also relatively common in patients with sick sinus syndrome, atrioventricular block and CAD. In patients with cardiac amyloidosis, impairment of the chronotropic response has been increasingly recognized. In a recent multicentre cohort study, chronotropic incompetence was prevalent and significantly correlated with reduced exercise capacity, suggesting that analysis of exercise-induced HRR may contribute to the clinical assessment in this population [62]. 

The diagnosis of chronotropic incompetence should be considered when a subject fails to achieve 80% of age-predicted maximum HR. However, the traditional formula (220 − Age) is not suitable for patients with CV disease or individuals taking negative chronotropic medications. Consequently, it is recommended to use an equation derived from a population that closely resembles the target group (e.g., Tanaka et al. equation in apparently healthy persons, Brawner et al. equation in those with suspected CV disease) [8,61].

Another method for evaluating chronotropic response is to measure chronotropic index (Wilkoff method). It is calculated by dividing the HR response (defined as the difference between resting HR and the maximal HR achieved during maximal exertion) by the difference between resting HR and age-predicted maximum HR. A chronotropic index < 80% is indicative of chronotropic incompetence and predicts a poor prognosis [61].

A delayed recovery of HR after exertion is another parameter associated with an increased risk of CV events, all-cause mortality, and death, independently of the peak HR [63] and regardless of age, gender, exercise capacity and left ventricular systolic function [64]. The physiological basis of HR recovery has been described previously (Section 2). While many methods have been used to define HR recovery cut-off, an abnormal HR recovery is generally defined as a reduction from the peak HR of <12 bpm/min (or <18 bpm/min if recovery was “active,” e.g., unloaded cycling or slow walking) in the first minute of passive supine recovery and of <42 bpm after 2 min of recovery [65,66]. A recent meta-analysis showed that, for every 10 bpm/min decrement in HR recovery rate, the risk of CV events and all-cause mortality was increased by 13% and 9%, respectively. Moreover, it showed that 1 min and 2 min HR recovery rates were equally effective at predicting all-cause mortality while the 2 min HR recovery rate appeared more sensitive for predicting CV events [63].

#### 5.2.2. Blood Pressure Response

The BP response during EST can provide important diagnostic and prognostic information [67].

Current guidelines provide conflicting opinions regarding normal values of BP response to exercise: the American Heart Association recommends that maximal systolic BP should not exceed 210 mmHg in men and 190 mmHg in women, whereas the European Society of Cardiology sets higher thresholds of 220 mmHg for men and 200 mmHg for women and the American College of Sports Medicine advises a universal systolic BP threshold of 225 mmHg, applicable to all genders [7,68,69]. Moreover, the applicability of these guidelines to highly trained athletes is not clearly defined. To address this gap, Caselli et al. [22] derived athlete-specific reference values from a large cohort of elite athletes, identifying upper systolic BP thresholds of 220 mmHg in men and 200 mmHg in women and diastolic BP upper limits of 85 mmHg in men and 80 mmHg in women. Interestingly, the small subset of healthy athletes with BP values above the 95th percentile included those with superior physical performance, endurance training, and more pronounced cardiac remodeling [22].

Evidence from systematic reviews and meta-analyses highlights a complex and sometimes conflicting association between systolic BP responses to exercise and mortality [70]. Notably, in individuals with high fitness levels, a higher systolic BP during exercise is associated with greater external workload and a lower incidence of CV events [21]; by contrast, other investigations have shown that an exaggerated BP responses to exercise predicts the future onset of hypertension and may trigger atherosclerotic plaque rupture, potentially leading to acute CV events [71]. As a result, the use of an absolute threshold of peak systolic BP to define an abnormal BP response to exercise may be misleading, mainly in populations with higher fitness and low CV risk. In high-performance athletes, peak SBP values may exceed guideline-defined thresholds at extreme workloads, likely reflecting elevated cardiac output rather than pathological vascular response [70].

Consistently, recent data in endurance athletes have shown that peak SBP alone demonstrates modest diagnostic accuracy in identifying hypertension on 24 h ambulatory blood pressure monitoring, whereas workload-indexed parameters provide improved discrimination [72]. To overcome these limitations, workload-indexed metrics such as the SBP/MET slope have been proposed. This parameter reflects the increase in SBP relative to achieved metabolic demand. Hedman et al. [21] demonstrated that an SBP/MET slope > 10 mmHg/MET was associated with increased long-term mortality, whereas the prognostic significance of peak SBP varied according to baseline cardiovascular risk.

The SBP/MET slope is also a valuable index for assessing CV adaptation in elite athletes. In this population, an elevated SBP/MET slope (>6.2 mmHg/MET [21]) has been associated with increased left ventricular thickness (even in the absence of systolic/diastolic dysfunction), reduced exercise capacity and higher risk of developing arterial hypertension and mortality compared with their counterparts. Accordingly, an elevated SBP/MET slope may represent an early marker of long-term CV risk and the initial manifestation of a maladaptive cardiac response [73].

Moreover, workload-indexed parameters such as the SBP/workload ratio have also demonstrated prognostic value in patients with heart failure, further supporting the concept that indexing systolic BP to external workload may provide incremental risk stratification beyond peak SBP alone [74,75]. Consistent with previous analysis [76,77], systolic BP assessed at a predefined submaximal workload demonstrates superior prognostic value compared with peak systolic BP and more effectively identifies individuals at increased risk of CV disease.

In contrast to hypertensive responses, a drop in systolic BP by ≥20 mmHg despite increasing workload is a pathological finding. When accompanied by other evidence of ischemia, this finding is an absolute indication to terminate the EST. A sustained, exercise-induced decrease in peak systolic BP is predictive of poor prognosis and often related to evidence of severe, multivessel CAD. This phenomenon is best explained by acute left ventricular pump failure secondary to extensive myocardial ischemia [78]. The clinical significance is greatest when hypotension occurs at low workloads and is accompanied by other ischemic features (e.g., ST-segment depression or angina). Beyond ischemia, exercise-related hypotension may be observed in a variety of other clinical scenarios, including cardiomyopathy, LV outflow tract obstruction, enhanced vagal tone, hypovolemia, the use of antihypertensive medications, and arrhythmias.

### 5.3. Ventricular Pre-Excitation

Ventricular pre-excitation is a cardiac conduction disorder characterized by the persistence of an abnormal accessory pathway that conducts electrical impulses from the atria to the ventricles, bypassing the normal atrioventricular node-His bundle axis. The Wolff–Parkinson–White (WPW) syndrome refers to the presence of an overt accessory pathway in combination with symptomatic, usually recurrent, tachyarrhythmias [79]. During sinus rhythm, the typical electrocardiographic pattern of a ventricular pre-excitation in WPW syndrome includes: a short PR interval (≤120 ms); a slurring of the initial segment of the QRS complex (delta wave); and a wide QRS complex (>120 ms). Delta wave may be particularly evident in highly trained athletes who exhibit an increased vagal tone and prolonged atrioventricular node conduction time. Among patients with WPW, atrioventricular reentrant tachycardia (AVRT), either orthodromic or antidromic, is the most common arrhythmia, followed by atrial fibrillation.

**Figure 4:**
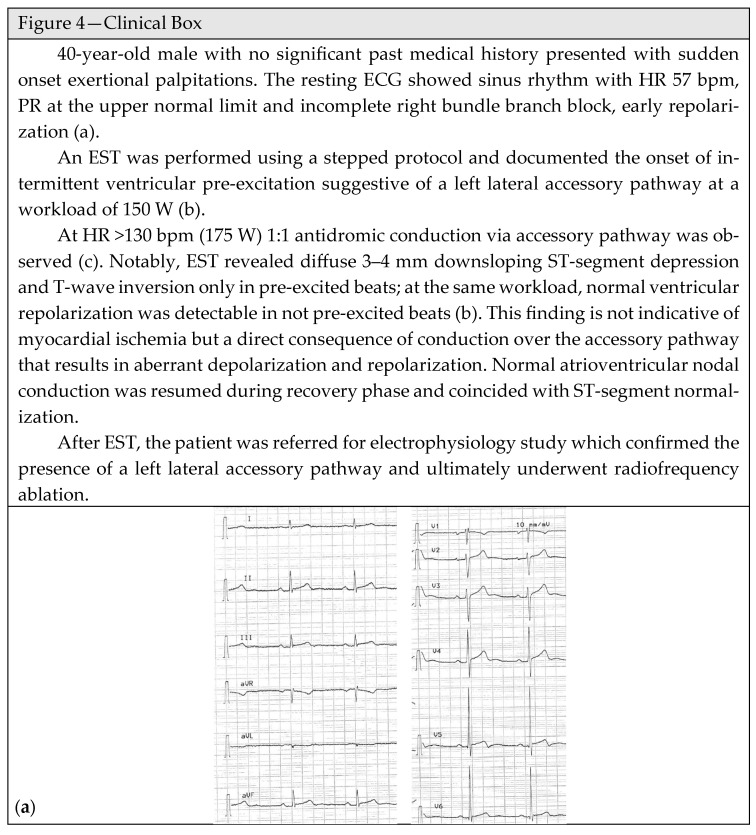

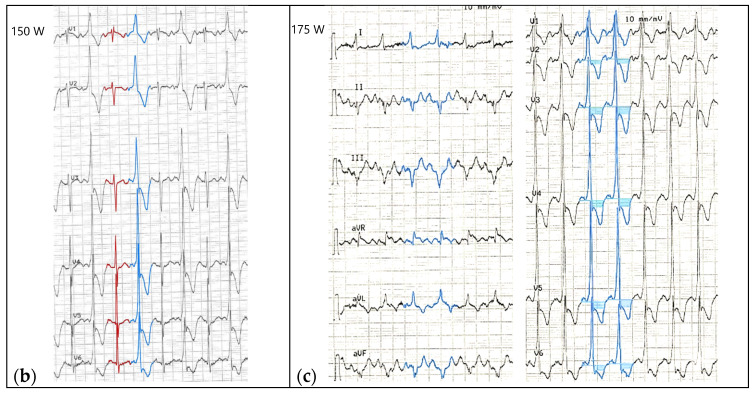
Clinical Box. (**a**) Resting ECG. (**b**) Intermittent ventricular pre-excitation (blue line) and normal atrioventricular conduction (red line). (**c**) Shows 1:1 antidromic conduction via accessory pathway (blue line) and secondary ST-segment depression (light blue area).

The capability of the accessory pathway to allow a rapid non-decremental atrioventricular conduction exposes subjects with WPW syndrome to an increased risk of malignant arrhythmic events and SCD, also in athletes [80] [81]. Consequently, a proper risk stratification is essential in these subjects, especially in athletes, to identify those with high-risk accessory pathways able to sustain rapid conduction and potentially leading to life-threatening ventricular arrhythmias (Figure 4—Clinical Box).

Current guidelines recommend performing an electrophysiological study in asymptomatic patients and professional athletes with ventricular pre-excitation. A non-invasive evaluation with EST should also be considered for asymptomatic subjects without high-risk occupations/hobbies [79].

The identification, during EST, of a sudden loss of ventricular pre-excitation, with the disappearance of the delta wave and complete normalization of the PR interval, is accepted as a low-risk predictor of malignant arrhythmias [82,83]. The disappearance of ventricular pre-excitation during exercise implies a long refractory period of the accessory pathway, even during adrenergic stimulation, identifying a low-risk pathway [84]. Evidence of an intermittent ventricular pre-excitation at rest is not necessarily indicative of a low-risk pattern, as exercise-dependent adrenergic stimulation can markedly improve accessory pathway conduction and refractoriness [85].

EST may provide additional diagnostic value by unmasking hidden accessory pathways; during the recovery phase, the physiological increase in vagal tone slows down the atrioventricular nodal conduction, making the conduction over the accessory pathway more evident. Moreover, evidence of multiple accessory pathways with different pre-excited morphologies on resting ECG or EST is defined as a marker of high risk.

Interpretation of EST in patients with ventricular pre-excitation may be challenging, leading to incorrect diagnoses and improper risk assessment. During exercise, sympathetic stimulation enhances atrioventricular nodal conduction and reduces the degree of ventricular pre-excitation, potentially leading to an apparent disappearance of the delta wave and a misdiagnosis of a low-risk accessory pathway. On the other hand, intermittent pre-excitation during exercise, with alternating presence and absence of the delta wave, could mimic other arrhythmic conditions such as atrial fibrillation or multifocal atrial tachycardia intermittent conduction [84]. Moreover, the accessory pathway causes abnormal ventricular activation and repolarization, responsible for ST-segment depression or T-wave inversion, which can be misinterpreted as ischemic changes. These electrocardiographic alterations, particularly in young healthy patients, are typically not associated with underlying CAD but may serve as an indication for the presence of an accessory pathway, even when a delta wave is not clearly visible (Figure 4—Clinical Box). Usually, the WPW-related ST-T alterations are deflected in the opposite direction with respect to the delta wave vector, the ST-segment change is nonhorizontal, and the T-wave inversion is nonsymmetrical. By contrast, when WPW syndrome coexists with an ischemic ST-T alteration, the ST-segment is horizontal, in accordance with the delta wave vector, the T-wave inversion is symmetrical and ST-T changes appear in two or more contiguous leads with angina symptoms [56].

To date, however, the role of EST for risk stratification in patients with ventricular pre-excitation remains controversial. This is because abrupt loss of ventricular pre-excitation during EST is observed in only a minority of patients, and the overall accuracy of EST in excluding high-risk accessory pathways appears to be moderate [83]. When EST does not allow confirmation of a low-risk accessory pathway, or when the presence of multiple accessory pathways is suspected, invasive evaluation with an electrophysiological study is recommended.

### 5.4. Ventricular Arrhythmias

PVBs are a common finding in the general population with a similar prevalence in sedentary subjects and athletes, and no substantial differences according to the training volume and sport type [86,87,88]. PVBs are observed during EST in approximately 5–10% of athletes, and their prevalence increases with age [89]. In most cases, ventricular arrhythmias occur in the absence of an underlying heart disease; however, in a minority of individuals, PVBs may represent the only manifestation of a pathological substrate at risk of SCD [90,91,92,93,94].

A careful evaluation of PVBs’ features and their behavior during EST is essential to accurately stratify the risk of pathological substrate, and the need for further investigations. Key aspects to assess include: PVB morphology, complexity and coupling interval, response to exercise, and reproducibility in different moments at the same type of examination or during a Holter ECG.

The assessment of the morphologic features of PVBs revealed during EST helps to identify the anatomical site of origin of the arrhythmia and infer the associated risk of an underlying cardiac disorder. Idiopathic PVBs usually occur in the absence of underlying structural heart disease and have a benign prognosis. The two most common sites of origin of idiopathic PVBs are the ventricular outflow tracts (“infundibular PVBs”) and the fascicular specialized cardiac conduction system. Infundibular morphology is characterized by a left bundle branch block (LBBB) pattern in V1 and inferior axis; the precordial transition beyond V3 identifies the origin from the right ventricle outflow tract while an earlier transition in V1–V2 denotes PVBs from the left ventricular outflow tract. Infundibular PVBs are usually monomorphic, appear in isolated beats and rarely in couplets or short runs of non-sustained ventricular tachycardia. This ventricular ectopy is usually very frequent during the day but typically decreases or disappears at peak of exercise and reappears during recovery [86,95]. Fascicular PVBs are another manifestation of benign idiopathic ventricular arrhythmias. The fascicular pattern is characterized by a typical right bundle branch block (RBBB) with a narrow QRS (<130 ms). A specific ECG pattern identifies PVBs with an origin from the posterior fascicle (RBBB and left anterior fascicular block morphology) or anterior fascicle (RBBB and left posterior fascicular block morphology). Both fascicular and infundibular PVBs generally arise from automatic ventricular foci.

**Figure 5:**
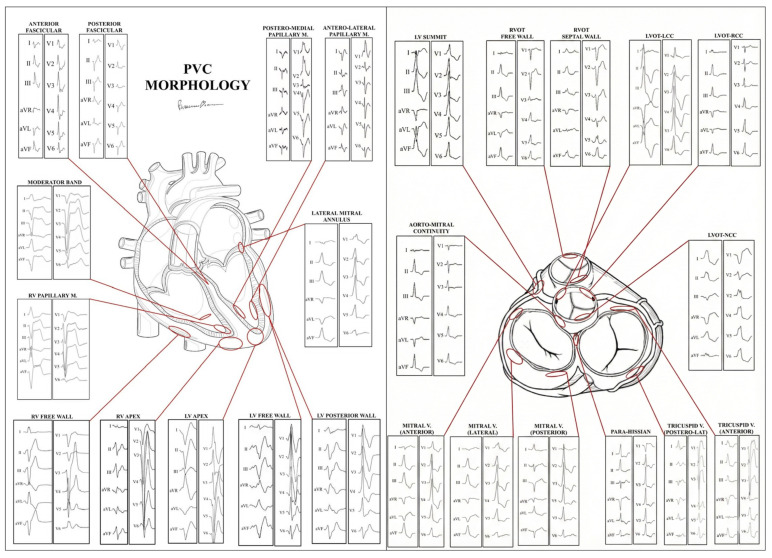
Premature ventricular beats morphology and probable site of origin. Schematic representation of the main sites of origin of PVBs and their ECG features. RV = right ventricle. LVOT = left ventricle outflow tract; NCC = non-coronary aortic cusp.

Ventricular ectopy originating from structures other than ventricular outflow tracts or fascicles displays different QRS patterns, is less frequent and should be carefully evaluated because it can to be associated with a cardiac disease [96]. Figure 5 schematically represents the main sites of origin of PVBs and their 12-lead electrocardiographic features. Among these, the most concerning pattern involves suggesting an origin from the lateral LV wall, the typical site of non-ischemic left ventricular scar (NILVS) [96]. A multicenter study by Muser et al. highlighted that PVBs with RBBB morphology and particularly those with superior axis (consistent with an origin from the inferolateral wall of the LV) were more frequently associated with myocardial abnormalities identified on cardiac magnetic resonance. Moreover, the presence of multifocal PVBs was associated with a pathological substrate and resulted in an independent predictor of a worse long-term outcome [97,98,99,100].

Calò et al. examined the electrocardiographic features of PVBs with RBBB morphology in apparently healthy athletes and demonstrated that PVBs exhibiting RBBB morphology, superior or intermediate axis and qR pattern in leads aVR and V1 are strongly associated with the absence of underlying structural disease [101]. Therefore, while PVCs with RBBB morphology and superior or intermediate axis often raise clinical concern, the additional presence of a qR pattern in aVR or V1 and a short intrinsicoid deflection time (<80 ms) suggests a favorable prognosis.

**Figure 6:**
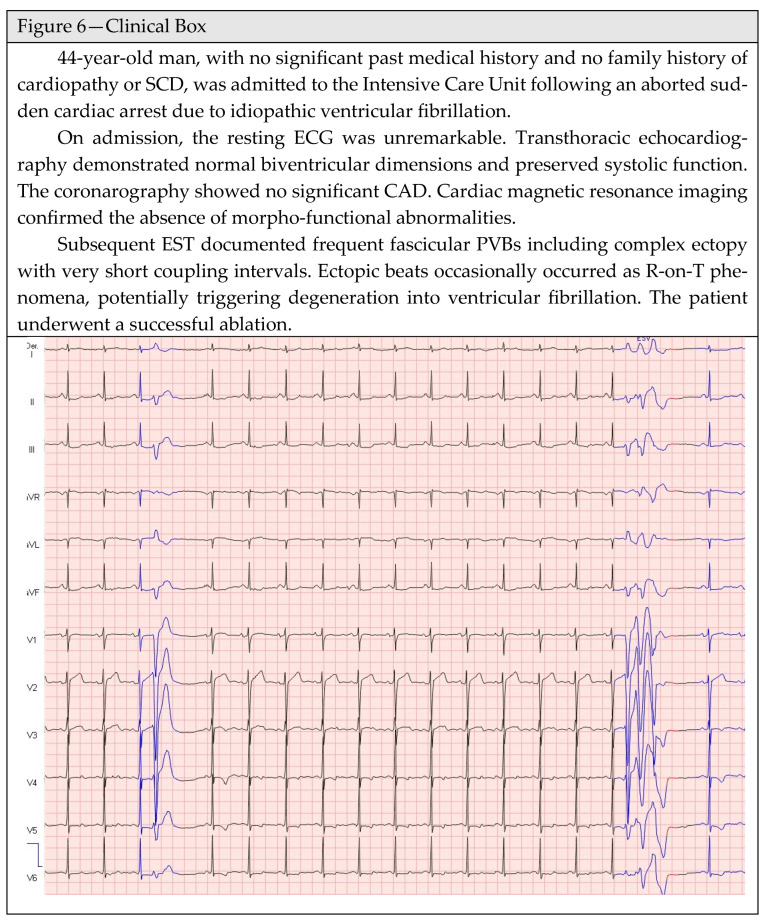
Clinical Box. In blue the fascicular premature ventricular beats realizing the R-on-T phenomenon, and organized in a triplet.

The complexity of PVBs, and hence the occurrence of couplets or runs of non-sustained ventricular tachycardia, may reflect the propensity of the arrhythmia to self-perpetuate, becoming sustained and potentially malignant. The evidence of short-couplet PVBs or R-on-T phenomena are warning signs for myocardial electrical instability which may predispose to malignant ventricular arrhythmia, independent from the PVB’s morphology [102] (Figure 6—Clinical Box).

EST is essential for evaluating PVBs response to exercise. Typically, PVCs that are present during the warm-up phase but are suppressed as the workload increases suggest a benign etiology. Conversely, ectopy that emerges or intensifies in frequency and complexity during high-intensity effort is consistently associated with an increased risk of structural heart disease [103].

Evaluating the PVBs’ reproducibility at repeated EST may facilitate risk stratification for underlying heart disease. Reproducibility is established when 3 or more PVBs with the same pattern and the same exercise-inducibility of a previous EST are recorded [92]. Therefore, when evaluating an individual with PVBs, particularly when isolated and not associated by other suspicious findings, it may be reasonable to repeat EST in order to improve risk stratification and to avoid unnecessary second-level investigations [96].

Corrado et al. proposed a simplified classification that distinguishes between “common” PVBs, generally idiopathic and benign, and “uncommon” PVBs, associated with higher likelihood of underlying heart disease. Common PVBs typically display infundibular or fascicular morphologies, often occur as isolated, are monomorphic and are suppressed by exercise. On the other hand, uncommon PVBs are characterized by RBBB morphology with a wide QRS complex (>130 ms) or LBBB morphology with a superior axis; “uncommon” PVBs are more frequently repetitive and polymorphic and tend to persist or increase in number and complexity with exertion. The evidence, during EST, of PVBs with “uncommon” characteristics should launch a cascade of CV evaluation in order to confirm or rule out the suspected cardiac pathology [102].

The following section outlines the diagnostic role of EST in specific conditions.

#### Catecholaminergic Polymorphic Ventricular Tachycardia

Catecholaminergic Polymorphic Ventricular Tachycardia (CPVT) is an inherited cardiac channelopathy characterized by life-threatening polymorphic ventricular arrhythmias adrenergically triggered. A missed diagnosis could lead to cardiac arrest during exertion.

CPVT is mostly associated with the mutation of cardiac ryanodine receptor (RyR2) or calsequestrin 2 genes (CASQ2). Other pathogenic variants involved CALM1, CALM2, CALM3, KCNJ2, TECRL and TRDN genes. These mutations resulted in the dysregulation of the intracellular calcium homeostasis, predisposing to triggered-activity polymorphic ventricular arrhythmias favored by adrenergic stimulation. Genetic testing identifies a pathogenic variant in almost 60–70% of probands [103].

The diagnosis of CPVT is often challenging, as this condition is typically associated with a normal resting ECG and structurally normal heart; therefore, the EST (or a Holter ECG with an exercise session) is the only diagnostic tool able to detect this condition [104].

The hallmark of CPVT is the onset of ventricular ectopy during EST. PVBs progressively increase in number and complexity as HR rises, evolving from isolated monomorphic PVBs to PVBs in bigeminy or couplets to bidirectional or polymorphic ventricular tachycardia, which may degenerate into ventricular fibrillation if exercise is not promptly discontinued, and the disappearance of the arrhythmias when the HR decreases (an on/off mechanism) [104]. Moreover, in patients with CPVT, ventricular arrhythmias are usually reproducible in different ESTs and elicited at the same HR.

EST plays a pivotal role in the follow-up of patients with established CPVT. It is essential for assessing the efficacy of ongoing medical therapy, optimizing drug choice and titration, and identifying patients who are refractory to combination medical therapy who may be candidates for ICD implantation or cardiac sympathetic denervation [105]. Furthermore, EST allows the identification of the HR at which ventricular arrhythmias are reproducibly induced, which may serve as a pragmatic reference to guide individualized exercise recommendations. However, this value should not be interpreted as a definitive safety threshold, as arrhythmic events may occur at lower intensities depending on adrenergic fluctuations and individual susceptibility.

### 5.5. Long QT Syndrome

Long QT Syndrome (LQTS) is an inherited cardiac channelopathy, characterized by prolongation of the QT interval and by a predisposition to ventricular arrhythmias and SCD [106]. LQTS is caused by pathogenic variants in multiple genes encoding cardiac ion channels. Each genetic subtype is associated with a relatively characteristic clinical phenotype and a distinct pattern of electrocardiographic features [106].

The upper normal value limits of the of QTc (with correction for HR according to Bazett’s formula) are 440 ms in men and 460 ms in women, and 480 msec in male athletes [107].

In LQTS, QTc prolongation often accompanies specific and bizarre morphologic changes in ventricular repolarization that may help predict the genotype. Notched T-waves are common in patients with LQTS type 2 and serve as an arrhythmic risk marker due to early after-depolarization; T-wave alternans is also a red flag of electrical instability, which can precede the onset of malignant ventricular arrhythmias usually triggered by adrenergic stimulation.

LQTS is easily diagnosed when a clear QTc prolongation of ≥ 480 ms is accompanied by syncope. However, up to 25% of “silent” mutation carriers have a normal resting QTc because of low penetrance and the dynamic nature of QT prolongation [108]. Therefore, in asymptomatic patients with borderline resting ECG features, EST and diagnostic scoring systems (e.g., LQTS Diagnostic Criteria [109]) could aid in identifying this condition.

In patients with normal or borderline resting QTc and suspected LQTS, EST is recommended to assess the QT behavior during active exercise and the recovery phase. During exercise and early recovery, QT measurement should be performed in the lead with the clearest T-wave morphology, typically lead II or lateral precordial leads (e.g., V5), avoiding leads with prominent U waves or significant motion artifacts. At rest, QT correction using Bazett’s formula remains widely adopted in clinical practice; however, during exercise and recovery, when the HR is >90 bpm, Fridericia’s correction may provide a more reliable estimate due to reduced heart rate-related overcorrection [110]. It was demonstrated that a prolonged QTc >480 ms during late recovery (the 4th minute after exercise) is a highly specific predictor of LQTS and a more sensitive marker than resting QT prolongation. Notably, a lower cut-off of 445 ms yields reduced specificity (approximately 90%) but increased sensitivity (approximately 90%) for distinguishing mutation carriers from non-carriers [33,109,111]. Therefore, the combination of resting QTc with QTc at 4 min recovery could predict a positive genetic result in subjects with suspected LQTS and borderline features.

Although exaggerated QTc prolongation during exercise is characteristic of LQTS and a robust predictor of LQT1, its utility is only modest, perhaps reflecting the technical difficulty of measuring QT accurately at peak exercise [111].

However, EST can provoke characteristic T-wave abnormalities at peak exercise, thereby serving as a valuable tool to unmask this diagnostic feature. A recent analysis conducted by Boeri et al. demonstrated that the evidence, at peak exercise of EST, of a complete fusion of the T- and P-wave (TP-fusion) in all precordial leads except V1 predicted an 88% probability of being affected by LQTS even in subjects with normal baseline QTc [112]. This novel variable is defined when a broad positive T-wave ends just before the onset of the QRS deflection, completely incorporating the P-wave. TP-fusion appeared concomitantly with an increased peak HR (>145 bpm) and longer peak QTc (>450 ms) in all LQTS genotypes and, interestingly, in 55% of patients with genotype-positive and baseline QTc < 450 ms, highlighting the usefulness of TP-fusion as a marker of “likely LQTS” in otherwise normal or borderline subjects [112].

### 5.6. Conduction Disorders

#### 5.6.1. Sinoatrial Node Dysfunction and Atrioventricular Block

In selected patients presenting with exercise-related symptoms or resting second-degree Mobitz type I atrioventricular (AV) block at rest, EST is recommended to clarify the diagnosis [107,113,114]. Furthermore, EST is valuable for differentiating exercise-induced symptoms associated with chronotropic incompetence from those resulting from conduction disorders.

Under physiological conditions, an enhanced vagal tone, as observed during sleep or pain, leads to slower conduction through the AV node and decreased pacing rate of the sinoatrial node; by contrast, adrenergic activation, such as that induced by exercise, shortens the effective refractory period and increases conduction velocity as the sinoatrial node pacing rate increases. On the surface ECG, this physiological facilitation manifests as a shortening of the PR interval as the HR increases.

Therefore, in patients with Mobitz 1 or 2:1 AV block, EST can be valuable for non-invasively identifying the site of the conduction disorder. Improvement or resolution of AV conduction abnormalities during exercise, due to increased sympathetic activation, typically indicates a supra-hisian block that usually does not require intervention. By contrast, a worsening of AV block during exercise and the development of tachycardia-related exercise-induced second-degree or complete AV block suggest an infra-hisian disease that predicts progression to permanent AV block [113,115]. Patients with infra-hisian AV block often show intraventricular conduction abnormalities on resting ECG, but a normal resting ECG has also been reported. In such cases, EST should be performed with caution and in centers equipped for advanced cardiac monitoring and immediate management of high-grade AV block.

While uncommon in patients with normal AV conduction at rest, exercise-induced second-degree AV block can lead to exercise intolerance, sometimes requiring a PM implantation. This condition could be associated with congenital heart disease or underlying myocardial ischemia [116].

In athletes who participate in high-volume endurance sports, sinus bradycardia (even extreme), moderate prolongation of the PR interval, and first- or second-degree Mobitz type I AV block are traditionally considered physiological and reversible phenomena, usually disappearing with increased HR [117]. The main mechanisms behind this are the training-related increase in vagal tone and a decrease in the intrinsic pacemaker rate [118,119], even if some epigenetic mechanisms have been proposed. When sinoatrial node dysfunction is suspected, a short detraining period may be necessary to differentiate it from physiological bradycardia induced by exercise [107].

However, some individuals with a long history of sports activity remain bradycardic even after stopping training. This may be due to two additional factors: stress-related damage to the sinoatrial and AV tissues and post-transcriptional downregulation of ion channel genes influencing AV and sinoatrial node activity. In athletes or former athletes with bradycardia and AV block at rest, EST is useful to determine whether the conduction disorder is a training adaptation or indicates a pathological condition. Recent data indicate that intense physical activity, mainly endurance training, can speed up the development of sinus node dysfunction and AV node dysfunction [119,120].

#### 5.6.2. Bundle Branch Block

In patients with left bundle branch block (LBBB), EST can be useful to evaluate functional capacity and chronotropic response to physical exercise, as well as the potential presence of exercise-induced ventricular arrhythmias. By contrast, the assessment of ventricular repolarization abnormalities is compromised by LBBB, making the evaluation of ST-T alterations less accurate. Therefore, in patients with LBBB in whom ischemic heart disease is suspected, stress testing with imaging may be considered [91].

Exercise-induced LBBB is a rare phenomenon, with a prevalence of approximately 0.5–1%. Exercise-induced LBBB has distinct prognostic implications depending on the HR at onset [121,122]. Vasey et al. found no coronary disease in patients who developed exercise-induced LBBB at a heart rate > 125 bpm, whereas the incidence of coronary artery disease was significantly higher when the LBBB developed at lower HR [123].

In patients without structural heart disease, exercise-induced LBBB is attributed to rate-dependent aberrant conduction due to delayed recovery of LBB. As HR increases with exercise, electrical impulses reach the proximal conduction system before the fascicle has fully repolarized, thereby precipitating bundle branch block. In a subset of patients, exercise-induced LBBB can lead to exertional intolerance characterized by atypical chest pain and palpitations upon exceeding the threshold of onset. In this setting, EST is essential to correlate the patient’s exertional symptoms with the onset of the conduction disturbance, to assess repolarization abnormalities before the development of LBBB and to identify the HR of LBBB appearance. It was reported that regular training shortens the repolarization phase 3 of the action potential, thereby increasing the rate at which LBBB occurs and improving exertional intolerance [122].

Exercise-induced right bundle branch block (RBBB) is an infrequent finding during EST. Similarly to exercise-induced LBBB, it is caused by a frequency-dependent delayed recovery of RBBB and is not related to an increased risk of cardiovascular events [117].

### 5.7. Assessment of Cardiac Implantable Electronic Device Function During Exercise

Exercise testing plays a crucial role in optimizing pacing responses during exercise and identifying the mechanisms of exercise intolerance. The optimal strategy involves performing EST while analyzing the electrogram on the CIED programmer to detect possible exercise-related device dysfunction and verify the effectiveness of any parameter reprogramming. In pacemaker-dependent patients, the chronotropic incompetence is the main cause of exercise intolerance. Rate-responsive pacing (RRP) was developed to improve exercise capacity in patients with chronotropic incompetence. This pacing mode is particularly important in patients with HF, in which the exercise-related increase in cardiac output is strongly dependent on HR, because of reduced stroke volume. Inadequate rate-adaptive settings may lead to persistent chronotropic incompetence, contributing to the worsening of functional capacity. Conversely, an excessive increase in HR may result in an improper rise in oxygen demand, ischemia, and a worsening of HF. Standard settings for RRP do not adequately mimic physiological sinoatrial node behavior; therefore, it is recommended to tailor CIED setup, mainly in highly active patients, using a meticulous approach with simultaneous maximum-effort exercise and device monitoring [124]. Assessment of the RRP profile during EST is essential to verify whether the chronotropic response is appropriate and to guide the modification of CIEDs parameters such as accelerometer response factor, accelerometer reaction time and recovery time, maximum tracking rate, or medical therapy optimization [124].

Based on cardiopulmonary exercise testing and pacemaker stress echocardiography, Serova et al. introduced a novel algorithm for optimal RRP programming in patients with HF and demonstrated that, in selected patients, a tailored RRP optimization improves exercise tolerance, LV diastolic function, and quality of life [125].

Another CIED-dependent mechanism of exercise intolerance is the inadequate adaptation of the AV delay during exercise. Under physiological conditions, the PR interval shortens during exercise; this adaptation reduces atrial systole duration while extending diastolic filling time and increasing end-diastolic volume, stroke volume, and cardiac output. In modern CRT devices, dynamic AV delay optimization algorithms are developed to mimic this physiological response. By contrast, if a static AV delay is programmed and the intrinsic PR interval shortens below this value during exercise, intrinsic conduction will override, resulting in progressive loss of biventricular synchronization and, ultimately, complete loss of effective left ventricular capture. It was demonstrated that an individualized device programming with dynamic AV delay improves left ventricular reverse remodeling and systolic function compared with biventricular pacing at fixed atrioventricular delay [126]. Therefore, assessment of CRT performance during EST allows clinicians to verify the appropriateness of dynamic AV delay settings, ensuring that optimal biventricular capture is maintained up to maximal HR.

**Figure 7:**
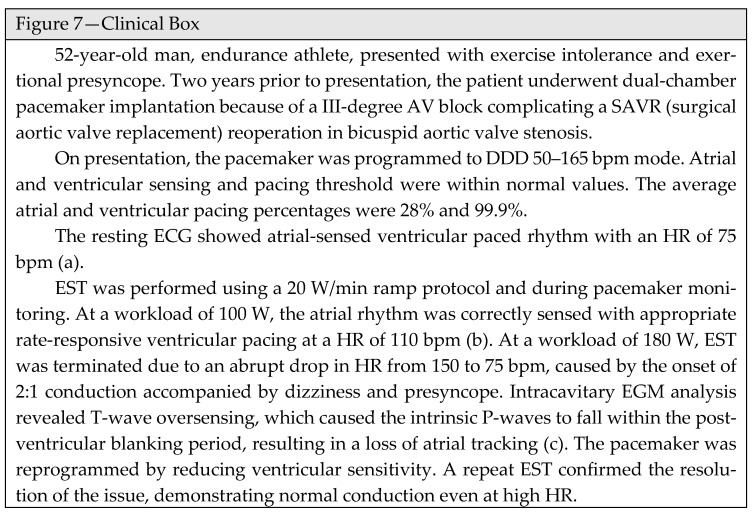

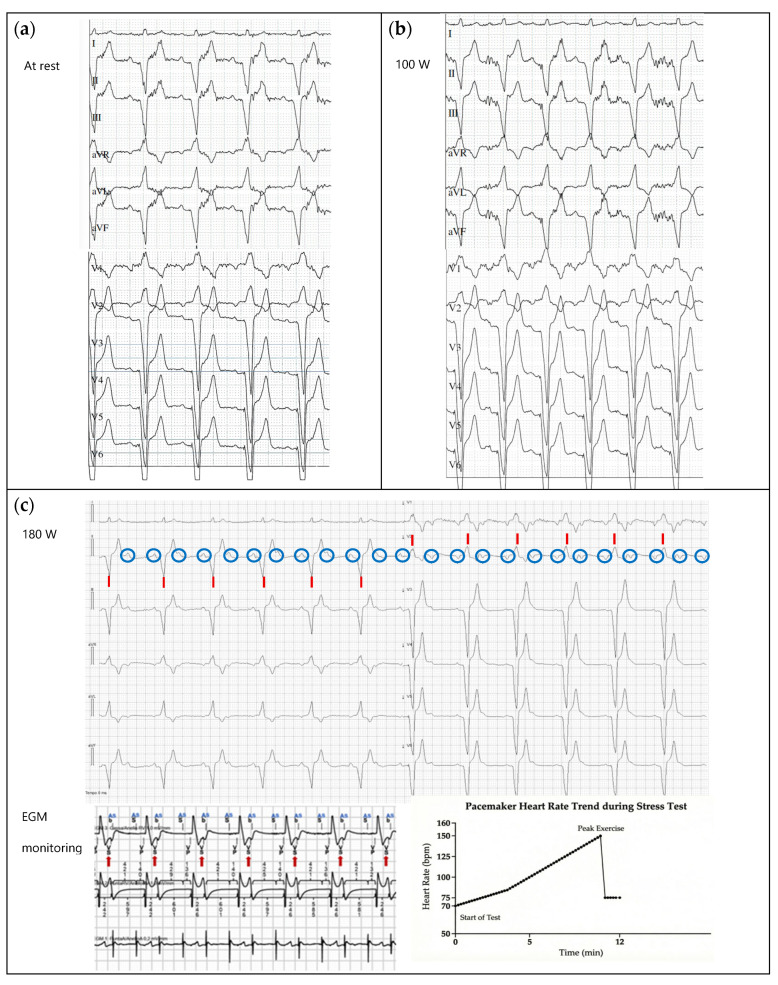
Clinical Box. (**a**) Atrial-sensed ventricular paced rhythm at rest. (**b**) Normal atrial tracking at 100 W workload. (**c**) T-wave oversensing (red arrow) resulting in loss of atrial tracking (2:1 behavior). P-waves are highlighted by blue circles; ventricular paced beats are indicated by red lines.

Additionally, in dual-chamber pacing mode, exercise intolerance may be attributed to the device’s “Wenckebach behavior”. As the atrial rate increases, ventricular pacing cannot exceed the programmed maximum tracking rate, resulting in progressively longer AV delay until a P-wave falls in the refractory period and is not tracked. However, if the total atrial refractory period is excessively prolonged and the intrinsic atrial rate exceeds the programmed maximum tracking rate during exercise, an abrupt 2:1 block may occur, resulting in a sudden slowing of the ventricular rate and exercise intolerance (Figure 7—Clinical Box). This condition is more frequent in young patients, in whom the physiologic increase in sinoatrial pacing rate during exercise requires a higher maximal tracking rate [127].

Moreover, exercise-induced augmentation of T-wave amplitude can result in T-wave oversensing and double-counting of the cardiac cycle. This may lead to the erroneous detection of a tachyarrhythmia, triggering inappropriate therapy delivery.

Therefore, EST is useful for verifying adequate sensing during exercise, particularly in young patients with subcutaneous implantable defibrillators, because the ability to discriminate ECG morphology is lower.

## 6. Limitations and Critical Considerations

Although the strengths and clinical applications of EST are well established, its limitations should be recognized. The diagnostic accuracy for obstructive CAD remains inferior to contemporary imaging techniques, and the interpretation of ECG changes may be influenced by baseline abnormalities, pharmacologic therapy, and training status. Moreover, heterogeneity in guideline-recommended cut-offs for blood pressure response, CI, and HRR reflects ongoing uncertainty regarding optimal thresholds, particularly in athletes. Finally, exercise-induced arrhythmias require cautious interpretation, as isolated findings may have low predictive value in otherwise healthy individuals. Therefore, EST findings should always be integrated within the overall clinical and demographic context.

## 7. Conclusions

EST is a widely available and cost-effective tool for evaluating hemodynamic and arrhythmic responses under physiological adrenergic stimulation. It provides integrated, dynamic data on functional capacity and exercise-inducible ischemia without the need for ionizing radiation or pharmacological stressors.

This review outlines practical guidelines (Table 3) regarding the execution and multifaceted applications of EST, integrating updated evidence with illustrative figures and exemplary clinical cases. Specifically, we explored the utility of EST in assessing BP response, advocating for a shift beyond the static cut-offs of current guidelines toward novel prognostic markers. Furthermore, EST remains pivotal in the evaluation of patients with brady- and tachyarrhythmias, and conduction disordes. Finally, in patients with CIEDs, EST represents a pragmatic tool for verifying the adaptation of device settings to real-life demands.

In summary, this review serves as an updated, accessible guide, underscoring the enduring and versatile role of EST in contemporary clinical practice.

## Figures and Tables

**Table 1:** Absolute and relative contraindications to exercise testing.

**Absolute Contraindications**
▪ Acute myocardial infarction within 2 days ▪ Hish-risk unstable angina ▪ Uncontrolled cardiac arrhythmia with hemodynamic compromise ▪ Active endocarditis ▪ Symptomatic severe aortic stenosis ▪ Decompensated heart failure ▪ Acute pulmonary embolism or pulmonary infarction ▪ Acute myocarditis or pericarditis ▪ Acute aortic dissection ▪ Acute pneumothorax ▪ Physical disability that precludes safe and adequate testing
**Relative Contraindications**
▪ Known obstructive left main coronary artery stenosis ▪ Moderate aortic stenosis with uncertain relation to symptoms ▪ Tachyarrhythmias with uncontrolled ventricular rates ▪ Acquired complete heart block ▪ Hypertrophic obstructive cardiomyopathy with severe resting gradient ▪ Recent stroke or transient ischemic attack ▪ Mental impairment with limited ability to cooperate ▪ Severe resting hypertension ▪ Uncorrected medical conditions, such as significant anemia, important electrolyte imbalance, and hyperthyroidism

Adapted from “Exercise standards for testing and training: a scientific statement from the American Heart Association” [7].

**Table 2:** Absolute and relative indications for terminating the exercise testing.

**Absolute indications for termination**
▪ ST elevation (>1.0 mm) in leads without Q waves due to prior MI (other than aVR, aVL, or V1) ▪ Drop in systolic BP of >10 mm Hg, despite an increase in workload, when accompanied by any other evidence of ischemia ▪ Typical angina ▪ Central nervous system symptoms (e.g., ataxia, dizziness, or near syncope) ▪ Signs of poor perfusion (cyanosis or pallor) ▪ Complex ventricular arrhythmias or other arrhythmias that interferes with normal maintenance of cardiac output during exercise ▪ Technical difficulties monitoring the ECG or systolic BP ▪ Patient’s request to stop ▪ Exaggerated hypertensive response (systolic blood pressure >250 mm Hg and/or diastolic blood pressure >115 mm Hg) *
**Relative indications for termination**
▪ Marked ST displacement (horizontal or downsloping of >2 mm) in a patient with suspected ischemia ▪ Drop in systolic BP of >10 mm Hg (persistently below baseline) despite an increase in workload, in the absence of other evidence of ischemia ▪ Increasing chest pain ▪ Fatigue, shortness of breath, wheezing, leg cramps, or claudication ▪ Development of bundle branch block that cannot be distinguished from ventricular tachycardia

Adapted from “Exercise standards for testing and training: a scientific statement from the American Heart Association” [7]. * BP thresholds should be interpreted within the clinical context. In highly trained athletes, SBP values above 250 mmHg may occur at very high workloads without clear evidence of acute risk; therefore, test termination should not rely solely on absolute BP values but also on symptoms and ECG findings. In patients with known aortic disease or heritable connective tissue disorders, exercise testing should be individualized and often limited to submaximal protocols with conservative BP limits.

**Table 3:** Practical summary of key parameters for exercise stress testing interpretation.

	Parameter	Normal Target	Abnormal Findings	Clinical Interpretation
**Test performance**	Exercise duration and effort scale (Borg RPE)	Borg RPE ≥ 17 Recommended duration 8–12 min	Symptom-limited test Test duration < 8 min or Borg RPE < 14	Reduced diagnostic reliability: consider repeat EST or alternative stress modality
**Functional capacity**	Peak workload (METs)	Predicted METs = 18 − 0.15 × age in males; 14.7 − 0.13×age in females	Peak METs < 85% of predicted values or marked reduction compared with prior testing	Strong predictor of cardiovascular events and all-cause mortality
**Chronotropic response**	Age-predicted maximum HR	220 − age; 208 − 0.7 × age (Tanaka et al. equation in apparently healthy persons); 164 − 0.7 × age (Brawner et al. equation in those with suspected cardiovascular disease)	Failure to reach ≥ 80% of predicted HR	Suggests autonomic dysfunction, sinus node disease, heart failure, or drug effect
	Chronotropic index	CI = HR response/(resting HR − age-predicted maximum HR) %	CI < 80%	Indicative of chronotropic incompetence and poor prognosis
	Heart Rate Recovery	≥12 bpm at 1 min (passive recovery); ≥42 bpm at 2 min	HR decrease < 12 bpm at 1 min (or <18 bpm with active recovery) or <42 bpm at 2 min → delayed HR recovery	Predictor of risk of cardiovascular events and all-cause mortality
**Blood pressure response**	Peak SBP; SBP/MET slope; SBP/W; SBP drop	SBP increase ≈ 5–10 mmHg/MET; no significant SBP fall	Exaggerated SBP response (≥220 men/≥200 women; guideline-dependent) Elevated SBP/MET slope (>10 mmHg/MET; >6.2 in elite athletes) SBP drop ≥ 20 mmHg (or >10 mmHg with ischemia), especially at low workload	Hypertensive response may indicate masked hypertension or maladaptive response to training; hypotension suggests severe CAD, LV dysfunction, or other cardiovascular pathology, and poor prognosis
**Ischemic ECG changes**	ST-segment deviation symptoms	No pathological ST-segment changes	Horizontal or downsloping ST depression ≥ 1 mm in ≥3 consecutive beats Slowly upsloping ST depression > 0.15 mV ST elevation ≥ 1 mm in ≥3 beats Persistence into recovery (>1 min)	Indicates inducible myocardial ischemia
**Ventricular Arrhythmias**	PVB morphology, complexity, response to exercise, reproducibility	Isolated, monomorphic PVBs; infundibular or fascicular (common) morphology; suppression or reduction with exercise	Uncommon morphology Polymorphic Complex Increase in frequency or complexity with workload R-on-T phenomenon; Reproducibility on repeated EST	Higher likelihood of structural heart disease or channelopathy; requires second-level investigations
**Conduction disorders**	AV and intraventricular conduction	PR interval shortens with exercise; stable AV conduction	Worsening AV block during exercise (new or advanced second/third-degree block); occurrence of bundle branch block	Suggests infra-Hisian disease or ischemia; may require EP evaluation or pacing.
**Disease-specific patterns**	QTc, pre-excitation, CPVT features	QTc shortens during exercise; abrupt loss of pre-excitation with increasing HR	QTc: QTc ≥ 480 ms at 4th minute recovery; peak TP-fusion. Persistence or appearance of pre-excitation during exercise or multiple accessory pathways → potentially high-risk. CPVT: progressive increase in PVB complexity and number as HR rises with suppression during recovery	Suggestive of LQTS, CPVT, or high-risk accessory pathway. Mandates targeted evaluation and therapy
**CIED assessment**	Rate response and AV delay (CIED electrocardiogram analysis during exercise)	Appropriate HR adaptation; preserved AV synchrony; stable ventricular tracking during exercise	Chronotropic incompetence despite RRP 2:1 tracking Loss of biventricular capture T-wave oversensing	Device-related exercise intolerance; requires reprogramming

AV, atrioventricular; bpm, beats per minute; CAD, coronary artery disease; CI, chronotropic index; CIED, cardiac implantable electronic devices; CPVT, catecholaminergic polymorphic ventricular tachycardia; ECG, electrocardiogram; EP, electrophysiological; EST, exercise stress testing; HR, heart rate; LQTS, long QT syndrome; LV, left ventricular; MET, metabolic equivalent; PVB, premature ventricular beat; QTc, corrected QT interval; RPE, rating of perceived exertion; RRP, rate-responsive pacing; SBP, systolic blood pressure.

## Data Availability

No new data were created.
